# The impact of COVID-19 management on the risk of exposure to specific chemical products: a multicentric Italian study based on 2017–2021 poison centers consultancies

**DOI:** 10.3389/fpubh.2025.1717130

**Published:** 2025-11-27

**Authors:** Felice Giordano, Lucrezia Lanciotti, Rosanna Maria Fidente, Leonello Attias, Valeria M. Petrolini, Carlo A. Locatelli, Giuseppe Bacis, Mariapina Gallo, Francesco Gambassi, Alessandra Ieri, Anna Lepore, Leonardo Pennisi, Francesco Coletta, Simone Esposito, Marco Marano, Mara Pisani, Lucilla Baldassarri

**Affiliations:** 1 National Center for Chemicals, Cosmetics and Consumer Protection, Istituto Superiore di Sanità, Rome, Italy; 2 Toxicology Unit, Pavia Poison Centre and National Toxicology Information Centre, Laboratories of Clinical and Experimental Toxicology, IRCCS Hospital of Pavia, Istituti Clinici Scientifici Maugeri, Pavia, Italy; 3 Bergamo Poison Control Center - ASST Papa Giovanni XXIII Hospital, Bergamo, Italy; 4 Medical Toxicology Unit and Poison Control Centre, Careggi University Hospital, Florence, Italy; 5 Poison Centre of Puglia, Policlinico Riuniti di Foggia, Foggia, Italy; 6 Emergency Anesthesia, Burn Intensive Care Unit and Poison Control Center, AORN Antonio Cardarelli, Naples, Italy; 7 Pediatric Poison Control Center, Children's Hospital Bambino Gesù, IRCCS, Rome, Italy; 8 General Pediatrics and Emergency Department 2nd Level, Bambino Gesù Children's Hospital, IRCCS, Rome, Italy

**Keywords:** poison control centers, poisoning, SARS-CoV-2 virus, hazardous substances, public health surveillance

## Abstract

**Introduction:**

In Italy, the emergency of COVID-19 led to the promotion of preventive measures like hygiene protocols, biocides use, and lockdowns. This study assesses whether the Italian COVID-19 prevention Decrees led to significant changes in exposure to specific chemicals, also examining the post-COVID-19 period (2021).

**Materials and methods:**

This multicentric observational study analyzed 2017–2021 exposure data to specific chemicals coming from six Italian Poison Centres (PCs). A multivariate analysis valued the association between the exposure to a specific product category and the predictive variables in different periods of 2020 and 2021, calculating crude and adjusted ORs and applying the Bonferroni correction for multiple comparisons.

**Results:**

The issuing of Decrees against COVID-19 conducted to peaks in exposure to chemicals. Higher proportions of children aged 1–5 were found among patients exposed to *Laundry detergents (PC-DET-1)* and Handwashing products (*Soaps* and *Gel/Spray*). For *Gel/Spray,* significant increases in the risk of exposure in 2020 (OR-lockdown period = 4.61; OR-post-lockdown period = 4.43) and 2021 (OR-whole year = 4.34) were observed. Adults (>19) account for higher percentages among patients exposed to *All-purpose products* (PC-CLN-2) and to *Surface Disinfectants* (PP-BIO-2). A significant increase was also found for PP-BIO-2 in lockdown period of 2020 (OR = 1.64). The risk of exposure to *Biocides for human* (PP-BIO-1) was almost statistically significant in post-lockdown period of 2020 (OR = 1.74).

**Discussion:**

This study evaluated the risk of exposure to chemicals during 2020 and 2021 vs. 2017–2019, considering the association between periods and exposures to specific chemicals as a crucial factor to correctly conduct these kinds of epidemiological studies.

## 1 Introduction

On December 31, 2019, Chinese health authorities reported a cluster of pneumonia cases of unknown origin in Wuhan, where zoonotic transmission from live animals to humans was initially suspected. Subsequent investigations by the Chinese Center for Disease Control and Prevention (CDC) identified the etiological agent as a novel coronavirus, later classified as Severe Acute Respiratory Syndrome Coronavirus 2 (SARS-CoV-2) by the Coronavirus Study Group of the International Committee on Taxonomy of Viruses (1). On January 30, 2020, the WHO Director-General declared the international SARS-CoV-2 outbreak a Public Health Emergency of International Concern (2). The virus exhibited a high transmission potential through multiple routes and posed significant challenges in terms of targeted therapeutic interventions. Following an assessment of its global spread and severity, WHO officially characterized COVID-19 as a pandemic on March 11, 2020 (3).

In response, worldwide health authorities implemented a range of non-pharmaceutical interventions to mitigate viral transmission, including physical distancing, mask usage, ventilation of indoor environments, lockdowns, surface cleaning and disinfection with products containing sodium hypochlorite, ethanol, or hydrogen peroxide (4), and hand hygiene. The U. S. CDC also advised frequent handwashing with soap and water or, when unavailable, the use of alcohol-based hand sanitizers containing at least 60% alcohol (5). These recommendations fed the fear of the emergency, leading to uncontrolled and often improper use of cleaning and disinfection products (6). Consequently, a global shift in exposure patterns to specific chemical agents was observed by worldwide Poison Centers (PCs) during 2020, as highlighted by Du Plessis et al. (Poisons Information Helpline of the Western Cape) (7) and Le Roux et al. (French PCs) (8).

In Italy, national authorities adopted several containment strategies. Initially, hygienic measures such as hand and surface disinfection using biocidal products were promoted (9, 10). The use of personal protective equipment, particularly face masks, was also recommended to reduce airborne transmission while preserving mobility in daily and occupational settings. As viral circulation persisted, more restrictive measures were introduced, including the suspension of non-essential work activities (10), culminating in a nationwide lockdown from March 10 to May 18, 2020 (11, 12). Additional decrees issued in the latter half of 2020 aimed to further limit interpersonal interactions (13–15).

Unlike other Italian studies published during COVID-19 period relying on data from individual PCs, this study evaluated data collected from six PCs. Its primary objective is to assess whether the periods following the implementation of Italian COVID-19 prevention decrees were associated with significant changes in the risk of exposure to specific chemical products. Furthermore, by including data from the post-COVID-19 period (2021), this study aims to evaluate whether the impact of the pandemic on chemical exposures persisted over time or whether the epidemiological profile of such exposures returned to pre-pandemic levels.

## 2 Materials and methods

### 2.1 Study design

This study is a multicenter, retrospective, observational analysis based on 2017–2021 data provided by six Italian PCs to the Italian National Institute of Health (Istituto Superiore di Sanità – ISS), concerning cases of exposure to chemical agents. PCs are facilities where expert operators support healthcare professionals and the general public in managing toxic effects caused by exposure to various agents (e.g., drugs, animal or plant toxins, chemicals, drugs of abuse). Although PCs do not operate under strict territorial jurisdiction, each center offers consultation services at the national level. Consequently, the dataset encompasses exposure and intoxication cases occurring throughout Italy.

A descriptive analysis was initially performed to characterize both exposed and unexposed individuals across each category examined. Subsequently, the analysis focused on identifying differences that distinguished the pandemic period from the preceding and subsequent ones.

### 2.2 Data sources

In accordance with scientific agreements established between ISS and PCs, the latter regularly transmit exposure data to the ISS. These data contribute to the National Informative System for the Surveillance of Dangerous Exposures and Poisonings to Chemicals (SIN-SEPI), serving the purpose of toxicovigilance (16).

The information provided by PCs are harmonized according to the Agreement of the Permanent Conference “Italian State-Regions,” February 28, 2008 (17), that defines rules for PCs activities and functioning. This information is not only useful in guiding the development of targeted therapeutic interventions for intoxicated individuals but also plays a critical role in monitoring exposures across vulnerable populations (such as the older adult, children, women, and occupational groups) over time and space. Such monitoring is essential for refining risk assessment approaches and for identifying Evidence-Based prevention strategies.

The geographical distribution of PCs in Italy is predominantly concentrated in the central and northern regions, with only two out of 10 centers located in the south (Naples and Foggia). Although PCs are not formally specialized in specific toxicological domains, some centers tend to manage particular types of exposures or respond to specific needs. For example, toxicological exposures in pediatric patients are particularly handled by the Children’s Hospital Bambino Gesù (CHBG)-Rome PC, drug administration during pregnancy and breastfeeding mainly pertain to Bergamo PC, the management of pesticide exposures is largely handled by Foggia PC (principally due to the closeness of extensive agricultural areas).

Despite the absence of a real-time telematic network or shared data entry and management software, PCs operate with the aim of collecting data as consistently as possible. This effort is intended to facilitate toxicovigilance activities and epidemiological studies that are comparable and aligned with scientific standards.

The Italian PCs collaborating in this study are the following:

- Toxicology Unit, Poison Centre and National Toxicology Information Centre, Istituti Clinici Scientifici Maugeri, IRCCS, Pavia;
- Poison Centre and Toxicology of Bergamo, ASST Ospedale Papa Giovanni XXIII, Bergamo;
- Toxicology Unit, Poison Control Center, Careggi University Hospital, Florence;
- Poison Centre of Puglia, AOU Policlinico Riuniti di Foggia;
- Poison Center, AORN Cardarelli Hospital, Naples;
- Pediatric Poison Control Center, CHBG, IRCCS, Rome.

### 2.3 Variables

The chemical products analyzed in this study were categorized using the EuPCS categorization system developed by ECHA (18), conceived to facilitate the transmission of information on the intended use of a mixture (according to Article 45 and Annex VIII of the Regulation (CE) 1,272/2008 (CLP)) (19) and to support the statistical analysis of poisonings at EU level.

Cosmetics, Waste products, Toys, Tobacco, and Self-defense Tools are chemical products not covered by CLP Regulation but managed by SIN-SEPI. All the exposures caused by such agents, except for Cosmetics *Handwashing Soaps* and *Gel/Spray products* (see Table 1), are included in “Other Chemical agents,” together with the rest of all EuPCS categories.

**Table 1:** Calls characteristics by specific chemical product (SIN-SEPI data from 2017 to 2021).

Variable	Cleaning products						Detergents				Biocides (BPR n.528/2012)				Handwashing cosmetics				Other chemical agents (OTH)		Total chemical agents	
	All-purpose (PC-CLN-2)		Bleaching (PC-CLN-3)		Floor/stone/ Tile (PC-CLN-12,13)		Laundry (PC-DET-1)		Dishwashing (PC-DET-3)		For human (PP-BIO-1)		For surfaces (PP-BIO-2)		Soap (COSM-HS)		Gel/Spray (COSM-HGS)					
	(N. 6,505)		(N. 11,059)		(N. 1,961)		(N. 3,560)		(N. 4,066)		(N. 1,801)		(N. 4,114)		(N. 408)		(N. 792)		(N. 22,079)		(N. 55,331)	
	*n.*	%	*n.*	%	*n.*	%	*n.*	%	*n.*	%	*n.*	%	*n.*	%	*n.*	%	*n.*	%	*n.*	%	*n.*	%
Gender																						
F	3,446	53.0	6,488	58.7	1,024	52.2	1,747	49.1	2,073	51.0	970	53.9	1,953	47.5	205	50.2	425	53.7	11,123	50.4	28,804	52.1
M	3,036	46.7	4,526	40.9	932	47.5	1,796	50.4	1,976	48.6	826	45.9	2,137	51.9	203	49.8	366	46.2	10,840	49.1	26,277	47.5
Unknown	23	0.4	45	0.4	5	0.3	17	0.5	17	0.4	5	0.3	24	0.6	-	0.0	1	0.1	116	0.5	250	0.5
Age Class (years)																						
<1	142	2.2	145	1.3	31	1.6	157	4.4	130	3.2	68	3.8	96	2.3	8	2.0	58	7.3	1,158	5.2	1,986	3.6
1–5	2,206	33.9	2,558	23.1	561	28.6	2,468	69.3	1,878	46.2	666	37.0	1,162	28.2	233	57.1	504	63.6	10,123	45.8	22,206	40.1
6–19	424	6.5	938	8.5	125	6.4	211	5.9	280	6.9	249	13.8	413	10.0	53	13.0	68	8.6	1,503	6.8	4,204	7.6
>19	3,584	55.1	7,190	65.0	1,214	61.9	683	19.2	1,698	41.8	790	43.9	2,358	57.3	111	27.2	152	19.2	8,866	40.2	25,871	46.8
Unknown	149	2.3	228	2.1	30	1.5	41	1.2	80	2.0	28	1.6	85	2.1	3	0.7	10	1.3	429	1.9	1,064	1.9
Circumstance																						
Non occupational	5,360	82.4	8,483	76.7	1,502	76.6	3,256	91.5	3,715	91.4	1,606	89.2	3,330	80.9	309	75.7	729	92.0	19,313	87.5	46,847	84.7
Occupational	223	3.4	251	2.3	88	4.5	6	0.2	80	2.0	33	1.8	317	7.7	1	0.2	5	0.6	704	3.2	1,664	3.0
Intentional	895	13.8	2,275	20.6	361	18.4	285	8.0	263	6.5	158	8.8	449	10.9	97	23.8	58	7.3	1,993	9.0	6,625	12.0
Unknown	27	0.4	50	0.5	10	0.5	13	0.4	8	0.2	4	0.2	18	0.4	1	0.2	-	0.0	69	0.3	195	0.4
Caller																						
Hospital	3,399	52.3	7,287	65.9	1,204	61.4	1,997	56.1	1,732	42.6	790	43.9	2,119	51.5	174	42.6	266	33.6	10,062	45.6	28,401	51.3
Extra-Hospital	3,069	47.2	3,735	33.8	747	38.1	1,547	43.5	2,313	56.9	1,009	56.0	1,985	48.2	234	57.4	526	66.4	11,952	54.1	26,735	48.3
Private citizen	2,623	40.3	2,861	25.9	581	29.6	1,335	37.5	1,988	48.9	844	46.9	1,606	39.0	207	50.7	472	59.6	10,373	47.0	22,589	40.8
Unknown	37	0.6	37	0.3	10	0.5	16	0.4	21	0.5	2	0.1	10	0.2	-	0.0	-	0.0	65	0.3	195	0.4
Route of exposure																						
Oral	4,480	68.9	7,086	64.1	1,602	81.7	2,877	80.8	3,591	88.3	1,549	86.0	2,360	57.4	372	91.2	669	84.5	15,753	71.3	39,870	72.1
Inhalation	846	13.0	2,736	24.7	195	9.9	60	1.7	51	1.3	43	2.4	1,054	25.6	6	1.5	10	1.3	2,186	9.9	6,685	12.1
Dermal	401	6.2	422	3.8	74	3.8	174	4.9	82	2.0	60	3.3	302	7.3	-	0.0	10	1.3	1,987	9.0	3,481	6.3
Ocular	501	7.7	513	4.6	42	2.1	394	11.1	129	3.2	68	3.8	343	8.3	4	1.0	33	4.2	1,195	5.4	3,210	5.8
Symptoms																						
No	2,702	41.5	3,088	27.9	777	39.6	1,656	46.5	2,314	56.9	1,050	58.3	1,397	34.0	257	63.0	594	75.0	11,332	51.3	24,982	45.2
Yes	3,777	58.1	7,910	71.5	1,175	59.9	1,893	53.2	1,739	42.8	744	41.3	2,708	65.8	151	37.0	196	24.7	10,668	48.3	30,133	54.5
Unknown	26	0.4	61	0.6	9	0.5	11	0.3	13	0.3	7	0.4	9	0.2	-	0.0	2	0.3	79	0.4	216	0.4
Period_years																						
2017–2019	3,801	58.4	6,394	57.8	1,243	63.4	2,207	62.0	2,386	58.7	966	53.6	2,178	52.9	232	56.9	168	21.2	13,389	60.6	32,369	58.5
2020	1,446	22.2	2,336	21.1	328	16.7	658	18.5	847	20.8	397	22.0	1,103	26.8	98	24.0	320	40.4	4,150	18.8	11,468	20.7
2021	1,258	19.3	2,329	21.1	390	19.9	695	19.5	833	20.5	438	24.3	833	20.2	78	19.1	304	38.4	4,450	20.6	11,494	20.8
Period_days																						
I (01/01–23/02)	849	13.1	1,513	13.7	289	14.7	455	12.8	526	12.9	241	13.4	435	10.6	62	15.2	64	8.1	3,136	14.2	7,442	13.4
II (24/02–10/03)	286	4.4	474	4.3	95	4.8	145	4.1	172	4.2	68	3.8	220	5.3	15	3.7	28	3.5	874	4.0	2,308	4.2
III (11/03–18/05)	1,297	19.9	2,278	20.6	373	19.0	649	18.2	865	21.3	382	21.2	763	18.5	92	22.5	166	21.0	4,366	19.8	11,021	19.9
IV (19/05–31/12)	4,073	62.6	6,794	61.4	1,204	61.4	2,311	64.9	2,503	61.6	1,110	61.6	2,696	65.5	239	58.6	534	67.4	13,703	62.1	34,560	62.5

According to the aim of the study, the product categories investigated were directly and indirectly affected by the measures implemented to contrast COVID-19 circulation. The products categorization was carried out by n.3 SIN-SEPI experts based on shared criteria. All variables included in the study underwent a quality check and were harmonized by the same experts from SIN-SEPI.

The variables were conceived as follows:

PC-CLN-2 - All-purpose (or multi-purpose) non-abrasive cleaners including degreasing agents (unless otherwise specified in other subcategories of cleaning products: Yes; No.

PC-CLN-3 - Bleaching products for cleaning or laundry use (excludes biocidal products): Yes; No.

PC-CLN-12 - Stone, tile and grout cleaning/care products: Yes; No.

PC-CLN-13 - Floor cleaning, care and maintenance products (excludes stone and tile): Yes; No.

PC-DET-1 - Laundry detergents: Yes; No.

PC-DET-3 - Dishwashing detergents: Yes; No.

PP-BIO-1 - Biocidal products for human hygiene: Yes; No.

PP-BIO-2 - Disinfectants and algaecides not intended for direct application to humans or animals: Yes; No.

Handwashing Cosmetics:

- soaps (COSM-HS): Yes; No.
- gel/spray products (excluding biocidal products – COSM-HGS): Yes; No.

Gender: Male; Female; Unknown.

Age class (years): <1; 1–5; 6–19; 19; Unknown.

Reason for exposure: Inadvertent (Non-Occupational, Occupational); Intentional; Unknown.

Caller: Hospital; Extra-Hospital, Extra-Hospital-Private citizen; Unknown.

Route of exposure: Oral; Inhalation; Dermal; Ocular.

Symptoms: Yes; No.

Period_years: 2017–2019; 2020; 2021.

Period_days: I: 01/01–23/02 (pre-pandemic); II: 24/02–10/03 (hygiene measures); III: 11/03–18/05 (restrictive measures – lockdown); IV: 19/05–31/12 (other restrictive measures – post-lockdown).

Years*Days: term of interaction (Period_years*Period_days).

The variable “Period_days” was drawn by identifying the period when Italian Ministerial Decrees/Circulars that recommended hygiene measures and/or imposed restrictive measures were issued in 2020.

The principal decrees are listed below:

**23-02-2020 to 10-03-2020**

*Hygiene measures*

Circular of the Minister of Health n. 0005443 of 22-02-2020 (9).

Decree 04-03-2020 (OJ no.55 of 04-03-2020) (10).

*Partial lockdown*

Decree 23-02-2020 (OJ no.45 of 23-02-2020) (some municipalities of 2 regions) (20).

Decree 25-02-2020 (OJ no.47 of 25-02-2020) (all municipalities of 6 regions) (21).

**11-03-2020 to 18-05-2020**

*Total lockdown*

Decree of 9 March 2020 (OJ no.62 of 09-03-2020) (national lockdown) (11).

*End of the total lockdown: some restrictive and hygiene measures survive*

Decree of 17 May 2020 (OJ no.126 of 17-05-2020) (12).

**19-05-2020 to 31-12-2020**

Starting from October 2020 several decrees limited interpersonal contacts, dividing the Italian territory into areas with different levels of infectious risk (14). The measures adopted by and within the Italian regions have been highly heterogeneous.

### 2.4 Selection criteria

To conduct this study, all 2017–2021 human exposures to chemical products managed by the six Italian PCs listed above were considered (n. 55,331). Analyses were performed after excluding cases with missing data (n = 1,372; 2.5%). Specifically, any record lacking information on at least one of Gender, Age class, or Circumstance of exposure, was excluded. This decision was supported by the observation of only minimal changes in ORs (see Supplementary Table S1). The number and percentage of excluded cases due to missing data on the total of exposures for each specific product category under investigation is reported below:

- PC-CLN-2: n.188; 2.9%
- PC-CLN-3: n.293; 2.6%
- PC-CLN-12,13: n.44; 2.2%
- PC-DET-1: n.61; 1.7%
- PC-DET-3: n.93; 2.3%
- PP-BIO-1: n.35; 1.9%
- PP-BIO-2: n.119; 2.9%
- COSM-HS: n.4; 1.0%
- COSM-HGS: n.10; 1.3%
- OTH: n. 549; 2.5%

It should be noted that the cumulative number of missing values across all product categories exceeds the total number of missing data. This discrepancy arises because subjects with unknown Gender, Age class, or Circumstance of exposure may have been associated with multiple product categories under investigation.

### 2.5 Statistical analysis

A seasonality analysis is reported in Supplementary Figure S1, showing a bar chart of the total number of exposures to chemical agents per month between 2017 and 2021. In 2020, the higher number of exposures compared to other years was observed between February and May, when a peak was reached (n. 1,255). A higher value was also observed in July 2020 (n. 1,164). Excluding 2020, the yearly peak was usually reached in June. From August to November the highest number of exposures was found in 2021.

The descriptive analysis considered the exposed and non-exposed subjects to each product category under investigation.

A multivariate analysis evaluated the association between the exposure to a specific product category (dependent variables) and the predictive variables (independent variables) by calculating the ORs (95% CI). Crude ORs are included in the Supplementary Table S2. The analyses intended to determine whether the potential risk factors “Period_years” and “Period_days” were associated to the dependent variables and, specifically, if the risk of exposure during 2020 (different periods) was higher than the reference (2017–2019). Additionally, a comparison with 2021 (post-COVID-19 period) was provided to evaluate a potential decrease in COVID-19-related exposures.

A logistic regression was applied to the multivariate analysis, correcting the potential risk factors for the possible confounders (Gender, Age class, Circumstance of exposure). Per each product-specific model the following covariates were included: Poison Center, Gender, Age Class, Circumstance of exposures, Period_years, Period_days, the interaction term Years*Days. For all variables the first category was set as reference. Considering multiple comparisons, the threshold used to declare statistical significance was *p =* 0.05/29 = 0.002 (Bonferroni correction). The goodness-of-fit was performed using the Hosmer & Lemeshow (H-L) test (a *p*-value>0.05 means that the model is a good fit while a p-value<0.05 indicates a poor fit).

Regression models were carried out through the following link function: logit (exposure to product category) = β_0_ + β_1_Age class+ β_2_Gender + β_3_Circumstance of exposure + β_4_Poison Center + β_5_Period_Years + β_6_Period_Days + β_7_(Period_Years * Period_Days).

Microsoft® Excel was used to perform graphs. IBM SPSS® Statistics software (ver.28) was used to conduct statistical analyses.

## 3 Results

**Figure 1:**
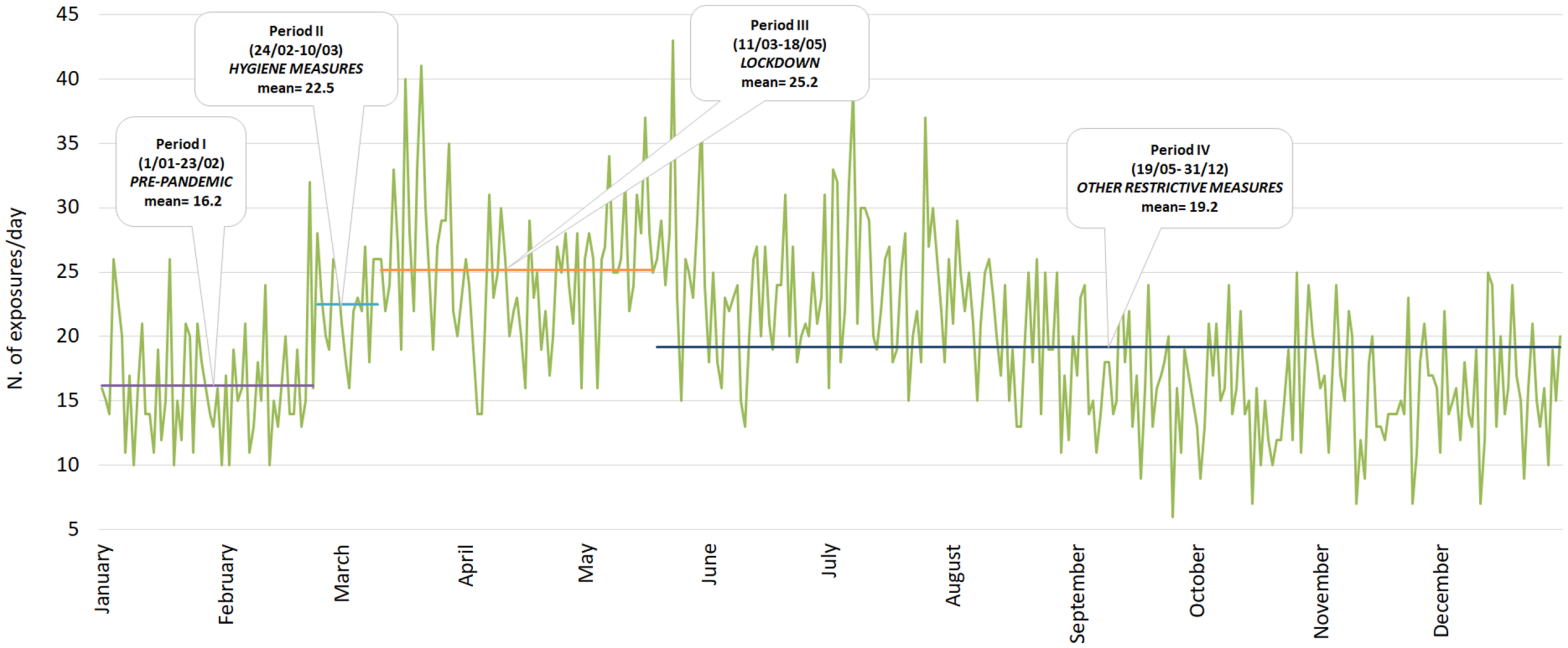
Daily exposures to chemicals and related mean of periods identified in 2020 by the issuing of ministerial decrees circulars (SIN-SEPI 2020).

In 2020, the issuing of Italian Ministerial Decrees/Circulars recommending hygiene measures and/or imposing restrictive measures immediately determined peaks in exposure to the chemical products in study (Figure 1). The highest mean value (25.2 n. exposures/day) can be observed for the lockdown period (Period III: from 11/03 to 18/05), followed by the mean value (22.5 n. exposure/day) observed during Period II (from 24/02 to 10/03) identified by the issuing of the first Circular recommending hygiene measures. During the post-lockdown period (Period IV: from 19/05 to 31/12) the mean seems to decrease (19.2 n. exposure/day). The other two Decrees, respectively issued on 24th of October 2020 and 18th of December 2020, do not constitute further cut-offs since the restrictions imposed refer to single feast days identifying a small number of exposures.

### 3.1 PCs distribution

The six PCs participating in the study managed a total of n. 55,331 consultations related to dangerous chemical exposures during the period 2017–2021 (Table 1). Specifically, the 56.5% (n.31,286) of consultancies was handled by Pavia PC, followed by Bergamo (17.3%; n. 9,592) and Florence (10.7%; n. 5,692). Lower percentages can be observed for Naples (6.3%; n. 3,506), Foggia (6.3%; n. 3,464) and CHBG-Rome (3.2%; n. 1,791) – data not shown.

**Figure 2:**
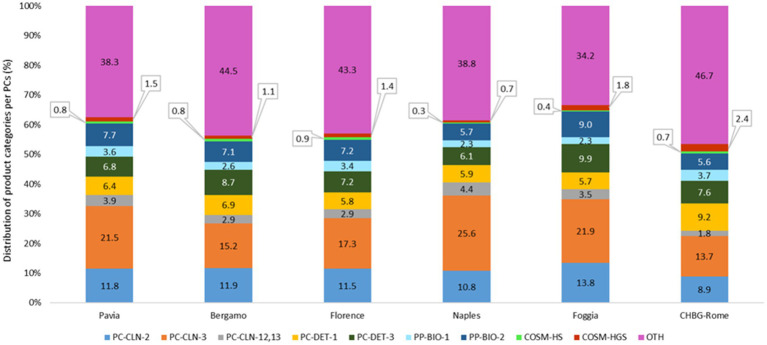
Percentage distribution of each specific product category by PC (SIN-SEPI data from 2017 to 2021).

When considering exposures to the specific products under investigation, percentage differences among PCs were observed (Figure 2): for *PC-CLN-2-Cleaning products*, Foggia PC managed a higher proportion of consultancies (13.8%; n. 478), while for *PC-CLN-3-Bleaching agents* and *PC-CLN-12,13-Floor/stone/tile products*, the PC of Naples recorded the highest percentages (25.6%; n. 898 and 4.4%; n. 156, respectively). The PC of CHBG-Rome showed the highest proportion of exposures for *PC-DET-1-Laundry detergents* and *COSM-HGS-Handwashing Gel/Spray*, with 9.2% (n. 164) and 2.4% (n. 43), respectively. The PC of Foggia also managed a greater proportion of consultancies related to *PC-DET-3-Dishwashing detergents* (9.9%; n. 342) and *PP-BIO-2-Surface disinfectants* (9.0%; n. 312).

### 3.2 Calls characteristics by chemical products

#### 3.2.1 Demographic variables

Among subjects exposed to the chemical categories showed in Table 1, the highest Females proportion was found among exposed to *PC-CLN-3-Bleaching products* (58.7%).

Age classes play an important role. In fact, subjects belonging to 1–5 age class show highest percentages of exposure among *PC-DET-1-Laundry detergents* and *COSM-HS/HGS-Handwashing Soaps and Gel/Spray Cosmetics* (69.3, 57.1 and 63.6%, respectively), while all cleaners and Biocides mainly involve subjects belonging to >19 age class (*PC-CLN-2-All-purpose* 55.1%, *PC-CLN-3-Bleaching products* 65.0%, *PC-CLN-12,13-Floor/stone/tile* 61.9%, *PP-BIO-1- Biocides for human* 43.9% and *PP-BIO-2-Surface disinfectants* 57.3%).

#### 3.2.2 Exposure variables

Non-occupational category takes a large proportion of exposures (*Total Chemical Agents* 84.7%) but among certain products this percentage reaches very high values (*PC-DET-1-Laundry detergents* 91.5%, *PC-DET-3-Dishwashing detergents* 91.4%, *PP-BIO-1-Biocides for human* 89.2% *COSM-HGS-Handwashing Gel/Spray* 92%). On the other hand, *PP-BIO-2-Surface disinfectants* have the highest percentage of Occupational exposures (7.7%). Intentional exposures mainly involve *PC-CLN-3-Bleaching* and *PC-CLN-12,13-Floor/stone/tile products*, as well as *COSM-HS-Handwashing soaps* (20.6, 18.4 and 23.8%, respectively).

*PC-CLN-3-Bleaching* and *PC-CLN-12,13-Floor/stone/tile products* have a high percentage of calls coming from hospital staff (*PC-CLN-3-Bleaching products* 65.9%, *PC-CLN-12,13-Floor/stone/tile products* 61.4% vs. *OTH-Other Chemical agents* 45.6%). Private citizen callers are more common among exposures caused by Handwashing Cosmetics than *OTH-Other chemical agents* (*COSM-HS-Soaps* 50.7%, *COSM-HGS-Gel/spray* 59.6% vs. *OTH-Other chemical agents* 47%).

Generally, the oral route is the most common way of exposure (*Total Chemical Agents* 72.1%) while Inhalation shows the highest proportion among exposures to *PC-CLN-3-Bleaching products* (24.7%) and to *PP-BIO-2-Surface disinfectants* (25.6%).

Chemical products showing the highest percentages of symptomatic exposures were *PC-CLN-3-Bleaching products* (71.5%) and *PP-BIO-2-Surface disinfectants* (65.8%). Considering 2020, the highest percentage of exposures was registered for patients exposed to *COSM-HGS-Handwashing Gel/spray* (40.4%); a similar value was recorded in 2021 (38.4%).

### 3.3 Exposure profile by chemical products

Table 2 shows the exposure profile to specific chemical products, directly (*PC-CLN-2-Cleaning product, PP-BIO-1-Biocides for human* and *PP-BIO-2-Biocides and COSM-HS/HGS-Handwashing soaps and gel/spray*) or indirectly (*PC-DET-1-Laundry* and *PC-DET-3-Dishwashing detergents*) affected by containment measures implemented to fight the COVID-19 pandemic. The variables “Period_years” and “Period_days” respectively refer to the exposure risk across the entire years (2020 and 2021) and across specific periods along all years. The interaction term “Years*Days” highlights exposure trends segmented by each defined period in 2020 and 2021.

**Table 2:** Product-specific exposure profile during the different periods in study (SIN-SEPI data from 2017 to 2021).

Variable	Cleaning products			Detergents		Biocides (BPR n.528/2012)		Handwashing cosmetics	
	All-Purpose (PC-CLN-2) OR_adj_ (95%CI)	Bleaching (PC-CLN-3) OR_adj_ (95%CI)	Floor/stone/tile (PC-CLN-12,13) OR_adj_ (95%CI)	Laundry (PC-DET-1) OR_adj_ (95%CI)	Dishwashing (PC-DET-3) OR_adj_ (95%CI)	For human (PP-BIO-1) OR_adj_ (95%CI)	For Surfaces (PP-BIO-2) OR_adj_ (95%CI)	Soaps (COSM-HS) OR_adj_ (95%CI)	Gel/Spray (COSM-HGS) OR_adj_ (95%CI)
Goodness-of-fit (H-L) (p-value)	*p =* 0.637	*p <* 0.001	*p =* 0.158	*p =* 0.040	*p =* 0.068	*p =* 0.755	*p =* 0.139	*p =* 0.410	*p =* 0.057
Period_years									
2017–2019	1	1	1	1	1	1	1	1	1
2020	1.25 (1.05–1.50) *p =* 0.014	1.00 (0.86–1.17)	0.77 (0.56–1.07)	1.09 (0.85–1.39)	0.80 (0.62–1.02)	0.77 (0.53–1.12)	1.07 (0.83–1.38)	0.70 (0.34–1.45)	1.46 (0.69–3.12)
2021	0.97 (0.80–1.17)	1.00 (0.86–1.16)	0.90 (0.67–1.22)	0.85 (0.66–1.09)	0.98 (0.79–1.23)	1.37 (1.02–1.85) *p =* 0.040	1.08 (0.84–1.38)	0.98 (0.53–1.82)	**4.34 (2.51–7.60)** ***p <* 0.001**
Period_days									
1^st^ (01/01–23/02)	1	1	1	1	1	1	1	1	1
2^nd^ (24/02–10/03)	1.07 (0.88–1.31)	0.97 (0.83–1.15)	1.21 (0.90–1.62)	1.10 (0.86–1.42)	1.02 (0.80–1.29)	0.84 (0.57–1.22)	**1.66 (1.32–2.09)** ***p <* 0.001**	0.74 (0.36–1.53)	1.25 (0.55–2.82)
3^rd^ (11/03–18/05)	1.04 (0.92–1.18)	1.04 (0.94–1.15)	0.92 (0.75–1.12)	0.96 (0.82–1.13)	1.06 (0.91–1.23)	1.05 (0.84–1.30)	1.04 (0.88–1.23)	0.83 (0.54–1.27)	0.94 (0.53–1.66
4^th^ (19/05–31/12)	1.12 (1.01–1.25) *p =* 0.034	0.99 (0.91–1.08)	0.92 (0.77–1.08)	1.15 (1.00–1.32) *p =* 0.046	0.97 (0.85–1.10)	0.89 (0.74–1.08)	1.23 (1.07–1.42) *p =* 0.004	0.74 (0.52–1.06)	1.09 (0.68–1.74)
Years*Days									
2017–19 by1^st^	1	1	1	1	1	1	1	1	1
2020 by2^nd^	0.94 (0.66–1.34)	1.08 (0.80–1.46)	0.75 (0.39–1.43)	0.72 (0.43–1.19)	1.29 (0.81–2.03)	1.52 (0.76–3.05)	1.37 (0.92–2.06)	0.83 (0.15–4.54)	2.40 (0.73–7.91)
2020 by3^rd^	0.95 (0.76–1.19)	1.11 (0.92–1.34)	0.93 (0.61–1.42)	0.70 (0.51–0.97) *p =* 0.032	1.34 (0.99–1.80)	1.43 (0.91–2.24)	**1.64 (1.21–2.22)** ***p =* 0.002**	2.57 (1.09–6.09) *p =* 0.031	**4.61 (1.92–11.05)** ***p <* 0.001**
2020 by4^th^	0.80 (0.65–0.97) *p =* 0.024	0.97 (0.82–1.15)	0.93 (0.65–1.33)	0.75 (0.57–0.99) *p =* 0.039	1.29 (0.98–1.68)	1.74 (1.16–2.60) *p =* 0.007	1.40 (1.07–1.84) *p =* 0.015	1.88 (0.85–4.16)	**4.43 (2.01–9.78)** ***p <* 0.001**
2021 by2^nd^	1.10 (0.75–1.61)	1.17 (0.86–1.59)	0.74 (0.39–1.42)	1.07 (0.64–1.80)	1.00 (0.62–1.60)	0.94 (0.46–1.87)	0.58 (0.35–0.96) *p =* 0.034	1.38 (0.36–5.23)	0.83 (0.28–2.53)
2021 by3^rd^	1.04 (0.81–1.33)	0.93 (0.77–1.13)	0.83 (0.55–1.25)	1.30 (0.94–1.80)	0.95 (0.71–1.27)	0.90 (0.60–1.33)	1.01 (0.73–1.39)	0.91 (0.36–2.15)	1.56 (0.76–3.18)
2021 by4^th^	0.90 (0.74–1.11)	1.01 (0.86–1.19)	1.01 (0.72–1.41)	0.99 (0.75–1.31)	1.04 (0.81–1.33)	0.94 (0.67–1.31)	1.08 (0.82–1.41)	0.96 (0.47–1.93)	1.23 (0.67–2.25)

In bold the significant p-values after Bonferroni correction for multiple comparisons (threshold at *p =* 0.002). H-L: Hosmer & Lemeshow test. Covariates included per each product-specific model: Poison Center, Gender, Age class, Circumstance, Period_years, Period_days, The interaction term Years*Days.

*PP-BIO-1-Biocides for human* revealed an almost statistically significant increase in the exposure risk during period IV (19/05–31/12) of 2020 (OR_adjusted_ = 1.74 (1.16–2.60); *p =* 0.007). The regression model of *PP-BIO-2-Surface disinfectants* showed statistically significant increases in the risk of exposure during period II (24/02–10/03) of all years (OR_adjusted_ = 1.66 (1.32–2.09); *p <* 0.001) and period III (11/03 – 18/05) of 2020 (OR_adjusted_ = 1.64 (1.21–2.22); *p =* 0.002).

*COSM-HGS-Handwashing Gel/Spray* indicate statistically significant fourfold increases in the risk of exposure during period III (11/03 – 18/05) and IV (19/05–31/12) of 2020 (period III: OR_adjusted_ = 4.61 (1.92–11.05); *p <* 0.001); period IV: OR_adjusted_ = 4.43 (2.01–9.78); *p <* 0.001). These products showed a significant increase also during 2021 (whole year) (OR_adjusted_ = 4.34 (2.51–7.60); *p <* 0.001).

## 4 Discussion

This multicentric Italian study based on PCs data investigated variations in the exposure risk to specific chemical agents used in disinfection and cleaning COVID-19-related activities. The analysis focused on selected periods of 2020, following the implementation of restrictive and hygienic Decrees and Circulars aimed at curbing COVID-19 transmission, and compared them with the same periods in previous years to minimize any seasonal bias. Multivariate regression analyses were conducted, adjusting for potential confounders (Gender, Age class, and Circumstance of exposure). Additionally, the study assessed the exposure risk to the same chemical products during the post-COVID-19 period (2021). Giordano et al. (22) already discussed these kinds of associations, although the analysis in the previous study was based on a smaller sample size, did not include post-COVID-19 period, and the statistical method used did not allow estimating ORs.

Consistent with existing evidence (23, 24), the number of consultancies managed by Italian PCs is heterogeneous. For instance, in this study the Pavia PC handled the largest share of consultancies for exposures to chemical products. This center normally manages a substantial portion of cases nationwide. Over half of the consultancies received by the Pavia PC originate from regions outside its own. In contrast, the other PCs included in this study mainly operate at a regional level, resulting in a smaller catchment area (23, 24).

As already described by other AA (8, 22, 25), a slight increase in the exposure to *PC-CLN-2-All-purpose cleaning products* and *PC-CLN-3-Bleaching products* during the lockdown period (Period III: from 11/03 to 18/05) compared to the reference was observed, but ORs lose their statistical significance after adjusting them for the possible confounders (Supplementary Table S2).

*PC-CLN-12,13-Cleaning products for floor* and *PC-DET-1-Laundry detergents* seem not to be associated with lockdown period compared to the same months of the reference period (2017–2019). We found similar findings for *PC-DET-1-Laundry detergents* using data coming from Pavia PC (22). It seems consistent thinking that the risk of exposure to *PC-DET-1-Laundry detergents* is related to their use: in fact, due to confinement measures, during lockdown period people reduced the need of washing clothes for outdoor activities. Children aged 1–5 are more likely exposed to *PC-DET-1-Laundry detergents* (69.3%) compared to *OTH-Other chemical agents* (45.8%). This finding could be associated to a specific type of laundry detergents, the Liquid Laundry Capsules for washing machines, that resemble candies because of the bright colors and may thus attract children (26, 27). These exposures can lead to both oral and eye injuries, with ocular exposures coming from the splash of detergent that comes out of the bitten capsule (26, 27). In response to this phenomena, European regulations now require child-resistant, opaque packaging and tougher, repellent-infused capsule materials. Further studies are needed to assess the effectiveness of these safety measures (28).

Among biocides, *PP-BIO-1-Biocides for human* have a different risk profile with respect to *PP-BIO-2-Surface disinfectants*. The former showed an almost statistically significant risk only during the post-lockdown period (period IV: from 19/05 to 31/12), probably due to the recovery of social activities with more physical contact and therefore a greater need for protection during interpersonal exchanges. To this end, dispensers of hand disinfectants were placed at the entrance of public areas and workplaces as well as in trains, buses etc. The risk of exposure to *PP-BIO-2-Surface disinfectants* increased both during lockdown (period III: from 11/03 to 18/05) and post-lockdown (period IV: from 19/05 to 31/12), as the need to disinfect living environments remained a priority even after the confinement period. Considering the risk–benefit ratio, during the COVID-19 pandemic the frequent use of hard surface cleaners and disinfectants was strongly recommended. To note that, during 2020 the number of dossiers submitted by Companies to put new biocides on the Italian market increased dramatically, going from around 250 dossiers presented in the three-year period 2017–2019, to more than 500 dossiers between March and December 2020 (source data: ISS evaluation group of biocides). Such numbers are clear indicators of the widespread use of this specific class of chemicals. With the end of the pandemic, many studies started warning about the dangerous release of secondary chemicals (e.g., volatile organic compounds - VOCs) while using cleaners and disinfectants (29, 30). In this regard, Dindarloo et al. (31) conducted a descriptive-analytical study involving 1,090 participants, aimed at investigating patterns of disinfectant use during the COVID-19 outbreak and assessing associated adverse effects on public health. The study revealed that approximately 60% of participants reported mixing various substances (e.g., sodium hypochlorite and alcohol with water) at home to create disinfectant solutions. Such combinations may result in the formation of secondary compounds that are harmful to human health. Alarmingly, only 10% of respondents indicated that they followed appropriate procedures for chemical mixing, highlighting a concerning trend that not only poses health risks but also fails to ensure effective disinfection.

Exposures to handwashing cosmetics seem to be less hazardous than those to *OTH-Other chemical agents* (absence of symptoms for *COSM-HS-Handwashing soaps*: 63% and *COSM-HGS-Handwashing gel/spray*: 75% vs. *OTH-Other Chemical agents*: 51%). In fact, due to their intended use, cosmetic products are meant to be applied on the human body and, as such, the intrinsic hazard of these products should be lower than that of chemicals intended to be applied on surfaces. The risk of exposure to *COSM-HGS-Handwashing gel/spray* in 2020 is statistically significantly higher during lockdown (from 11/03 to 18/05) and post-lockdown (from 19/05 to 31/12) period. As a matter of fact, it is known that these products are particularly linked to COVID-19 pandemic, being used to prevent the virus spread (32). It is interesting to note that the risk of exposure to *COSM-HGS-handwashing gel/spray* remained significant also during 2021 (post-COVID-19 period), indicating that these products continued to be widely used by the general population. This persistence underscores the need for increased supervision by Competent Authorities to ensure their safe use. Notably, these products may pose serious health risks, particularly due to the potential presence of methanol, a highly toxic substance (33). In fact, methanol-related fatal exposures were reported by the Arizona Poison and Drug Information Center in 2020 (34). Another critical concern is the potential for these products to be mistaken for food items and attract children. Their colorful packaging and appealing scents may lead to accidental oral exposure among children (35).

Unlike previous studies (16, 22), this work included data from the majority of Italian PCs and compared cases recorded during the pandemic not only with the pre-pandemic baseline, but also with the post-2021 period. This approach aimed to determine whether the observed increase in potentially toxic exposures to these products reflects a lasting change in public habits induced by the pandemic, or rather a temporary phenomenon. The results obtained suggest that any public health recommendation, however well-intentioned, should be accompanied by messages promoting caution and correct use and could be an encouragement for Competent Authorities to increase awareness on specific topics (e.g., safe use of chemicals, keeping chemicals out of reach of children, reading labels carefully), such as those that can be found on WHO or CDC websites (36, 37).

This work also confirms that data from poison control centers reflect health and social events and demonstrates how such data can be valuable in health surveillance plans. To further improve the entire system and the quality of data, the SIN-SEPI and the Italian PCs are involved in a Recovery and Resilience Plan (RRP)- project entitled “The national system for the control and surveillance of Chemicals to protect public health” funded by the Italian Ministry of Health (38).

The methodological approach adopted in this study demonstrated its effectiveness in addressing the research aims. Most articles available in literature are descriptive studies focusing on the distribution of variables across different timeframes and typically using the years prior to 2020 as a reference (8, 22, 39–41). Only recently regression models have been developed to estimate trends in PCs exposure data (42). To conduct such analyses effectively, it is essential to first investigate the association between time periods and exposures to specific chemicals, to avoid random correlations that could lead to misguided preventive measures.

## 5 Limitations of the study

This study is certainly affected by some limitations.

First, even if the EuPCS categorization of products can be easily applied, for many agents it was not possible to assign an unambiguous categorization because the name reported was incomplete or because the intended use, a characteristic on which the EuPCS classification is based, is not known. Secondly, although data are standardized and categorized by SIN-SEPI experts based on shared criteria, differences in the interpretation of some data from various PCs databases are still possible.

It must be highlighted that the COVID-19 public health emergency required medical professionals, including medical toxicologists at PCs, to prioritize activities related to the containment and management of the virus. This shift in focus inevitably led to a reduced emphasis on routine operations. Consequently, potential under-reporting during the most critical phases of the pandemic should be considered when interpreting data.

The exposure trends to the product categories investigated should also be interpretated considering the number of sold products during a specific period. This data could not be retrieved for the purposes of this study.

Furthermore, this study does not report information on clinical assessment of exposure severities, except for the presence/absence of symptoms in Table 1. The severity of exposures (intended as the *Poison Severity Score* – *PSS* (43), is a variable available in PCs databases, but it remains highly inconsistent among PCs in terms of both interpretation and reporting. Efforts are currently underway to improve this aspect.

## Data Availability

The raw data supporting the conclusions of this article will be made available by the authors, without undue reservation.
