# Supplementary figures and images for: The impact of COVID-19 management on the risk of exposure to specific chemical products: a multicentric Italian study based on 2017–2021 poison centers consultancies
Source: Front Public Health. 2025 Nov 27;13:1717130. doi: 10.3389/fpubh.2025.1717130 (PMC12695805; doi:10.3389/fpubh.2025.1717130)

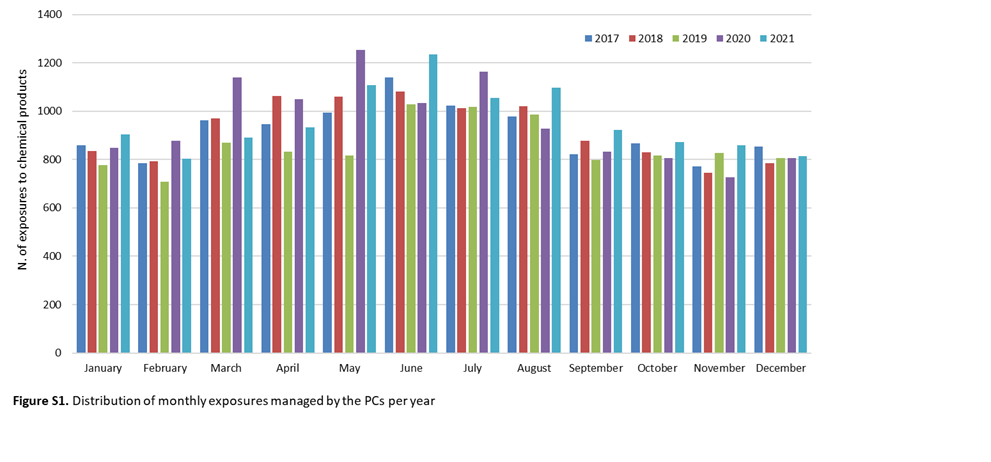

Supplement: Supplementary file 1 [file Image_1.tif]
