# Supplementary material for: The impact of COVID-19 management on the risk of exposure to specific chemical products: a multicentric Italian study based on 2017–2021 poison centers consultancies
Source: Front Public Health. 2025 Nov 27;13:1717130. doi: 10.3389/fpubh.2025.1717130 (PMC12695805; doi:10.3389/fpubh.2025.1717130)
Supplement: Supplementary file 2 [file Table_1.docx]

| **Table S1.** Comparison between Adjusted ORs with and without missing values (SIN-SEPI data from 2017 to 2021) | | | | | | | | | | | | | | | | | | |
| --- | --- | --- | --- | --- | --- | --- | --- | --- | --- | --- | --- | --- | --- | --- | --- | --- | --- | --- |
| **Variable** | **Cleaning products** | | | | | | **Detergents** | | | | **Biocides (BPR n.528/2012)** | | | | **Handwashing Cosmetics** | | | |
|  | **All-Purpose**  **(PC-CLN-2)**  OR_adjusted_  95%CI  *Missing values* | | **Bleaching**  **(PC-CLN-3)**  OR_adjusted_  95%CI  *Missing values* | | **Floor/stone/tile**  **(PC-CLN-12,13)**  OR_adjusted_  95%CI  *Missing values* | | **Laundry**  **(PC-DET-1)**  OR_adjusted_  *95%CI*  *Missing values* | | **Dishwashing**  **(PC-DET-3)**  OR_adjusted_  95%CI  *Missing values* | | **For human**  **(PP-BIO-1)**  OR_adjusted_  95%CI  *Missing values* | | **For surfaces**  **(PP-BIO-2)**  OR_adjusted_  95%CI  *Missing values* | | **Soaps**  **(COSM-HS)**  OR_adjusted_  95%CI  *Missing values* | | **Gel/Spray**  **(COSM-HGS)**  OR_adjusted_  95%CI  *Missing values* | |
|  | Yes | No | Yes | No | Yes | No | Yes | No | Yes | No | Yes | No | Yes | No | Yes | No | Yes | No |
| **Poison Center** |  |  |  |  |  |  |  |  |  |  |  |  |  |  |  |  |  |  |
| Pavia | 1 | 1 | 1 | 1 | 1 | 1 | 1 | 1 | 1 | 1 | 1 | 1 | 1 | 1 | 1 | 1 | 1 | 1 |
| Bergamo | **1.09**  **1.01-1.17** | **1.10**  **1.02-1.18** | **0.78**  **0.73-0.83** | **0.78**  **0.73-0.83** | **0.84**  **0.73-0.96** | **0.83**  **0.73-0.96** | 0.91  0.83-1.00 | **0.90**  **0.82-0.99** | **1.21**  **1.11-1.32** | **1.21**  **1.11-1.32** | **0.69**  **0.60-0.80** | **0.71**  **0.61-0.81** | 1.01  0.92-1.11 | 1.03  0.94-1.12 | 1.13  0.87-1.47 | 1.14  0.88-1.48 | **0.61**  **0.49-0.76** | **0.62**  **0.50-0.76** |
| Florence | 0.98  0.90-1.07 | 1.00  0.91-1.09 | **0.80**  **0.74-0.86** | **0.79**  **0.73-0.85** | **0.79**  **0.67-0.93** | **0.79**  **0.67-0.93** | 0.90  0.80-1.02 | 0.91  0.80-1.02 | 1.01  0.90-1.12 | 1.01  0.90-1.13 | 0.90  0.77-1.05 | 0.90  0.77-1.05 | 0.91  0.81-1.01 | 0.91  0.82-1.02 | **1.37**  **1.01-1.86** | **1.38**  **1.01-1.87** | 0.83  0.65-1.07 | 0.84  0.66-1.08 |
| Foggia | **1.18**  **1.07-1.31** | **1.21**  **1.09-1.34** | 1.04  0.95-1.13 | 1.04  0.95-1.13 | 0.93  0.77-1.13 | 0.90  0.74-1.10 | 0.95  0.82-1.11 | 0.96  0.83-1.12 | **1.43**  **1.27-1.61** | **1.45**  **1.28-1.63** | **0.60**  **0.47-0.75** | **0.59**  **0.47-0.75** | 1.12  0.99-1.27 | **1.13**  **1.00-1.28** | 0.58  0.33-1.01 | **0.53**  **0.30-0.96** | 1.07  0.82-1.40 | 1.09  0.83-1.43 |
| Naples | 0.89  0.80-1.00 | 0.90  0.80-1.01 | **1.24**  **1.14-1.35** | **1.23**  **1.13-1.34** | **1.20**  **1.01-1.43** | **1.23**  **1.03-1.46** | 0.96  0.83-1.12 | 0.97  0.83-1.13 | **0.86**  **0.74-0.99** | **0.85**  **0.73-0.98** | **0.60**  **0.47-0.75** | **0.60**  **0.47-0.75** | **0.70**  **0.60-0.82** | **0.69**  **0.59-0.81** | **0.46**  **0.26-0.82** | **0.46**  **0.26-0.83** | **0.36**  **0.24-0.54** | **0.34**  **0.23-0.52** |
| CHBG-Rome | 0.92  0.78-1.09 | 0.93  0.78-1.10 | 1.13  0.98-1.30 | 1.11  0.96-1.28 | **0.68**  **0.47-0.97** | **0.64**  **0.44-0.93** | 0.85  0.72-1.01 | 0.85  0.72-1.01 | 0.98  0.82-1.18 | 0.99  0.82-1.19 | 0.97  0.75-1.25 | 0.98  0.76-1.27 | 0.95  0.77-1.17 | 0.97  0.78-1.19 | 0.68  0.38-1.22 | 0.68  0.38-1.23 | 0.83  0.60-1.14 | 0.81  0.59-1.12 |
| **Period years** |  |  |  |  |  |  |  |  |  |  |  |  |  |  |  |  |  |  |
| 2017-2019 | 1 | 1 | 1 | 1 | 1 | 1 | 1 | 1 | 1 | 1 | 1 | 1 | 1 | 1 | 1 | 1 | 1 | 1 |
| 2020 | **1.26**  **1.06-1.50** | **1.25**  **1.05-1.50** | 0.99  0.85-1.14 | 1.00  0.86-1.17 | 0.77  0.56-1.06 | 0.77  0.56-1.07 | 1.11  0.87-1.42 | 1.09  0.85-1.39 | 0.80  0.63-1.02 | 0.80  0.62-1.02 | 0.85  0.59-1.24 | 0.77  0.53-1.12 | 1.05  0.82-1.35 | 1.07  0.83-1.38 | 0.70  0.34-1.45 | 0.70  0.34-1.45 | 1.62  0.78-3.37 | 1.46  0.69-3.12 |
| 2021 | 0.96  0.80-1.16 | 0.97  0.80-1.17 | 0.99  0.86-1.15 | 1.00  0.86-1.16 | 0.90  0.66-1.21 | 0.90  0.67-1.22 | 0.87  0.67-1.12 | 0.85  0.66-1.09 | 0.96  0.77-1.20 | 0.98  0.79-1.23 | 1.05  0.84-1.31 | **1.37**  **1.02-1.85** | 1.06  0.83-1.36 | 1.08  0.84-1.38 | 0.99  0.54-1.83 | 0.98  0.53-1.82 | **4.45**  **2.55-7.75** | **4.34**  **2.51-7.60** |
| **Period days** |  |  |  |  |  |  |  |  |  |  |  |  |  |  |  |  |  |  |
| 1^st^ (01/01 - 23/02) | 1 | 1 | 1 | 1 | 1 | 1 | 1 | 1 | 1 | 1 | 1 | 1 | 1 | 1 | 1 | 1 | 1 | 1 |
| 2^nd^ (24/02 - 10/03) | 1.09  0.90-1.32 | 1.07  0.88-1.31 | 0.98  0.84-1.15 | 0.97  0.83-1.15 | 1.18  0.88-1.58 | 1.21  0.90-1.62 | 1.10  0.86-1.42 | 1.10  0.86-1.42 | 1.01  0.80-1.28 | 1.02  0.80-1.29 | 0.85  0.59-1.24 | 0.84  0.57-1.22 | **1.60**  **1.27-2.01** | **1.66**  **1.32-2.09** | 0.74  0.36-1.54 | 0.74  0.36-1.53 | 1.25  0.55-2.84 | 1.25  0.55-2.82 |
| 3^rd^ (11/03 - 18/05) | 1.03  0.91-1.16 | 1.04  0.92-1.18 | 1.02  0.92-1.12 | 1.04  0.94-1.15 | 0.91  0.75-1.11 | 0.92  0.75-1.12 | 0.99  0.84-1.16 | 0.96  0.82-1.13 | 1.05  0.91-1.21 | 1.06  0.91-1.23 | 1.05  0.84-1.31 | 1.05  0.84-1.30 | 1.03  0.88-1.22 | 1.04  0.88-1.24 | 0.83  0.54-1.27 | 0.83  0.54-1.27 | 0.98  0.56-1.72 | 0.94  0.53-1.66 |
| 4^th^ (19/05 – 31/12) | **1.11**  **1.00-1.24** | **1.12**  **1.01-1.25** | 0.99  0.91-1.08 | 0.99  0.91-1.08 | 0.92  0.78-1.08 | 0.92  0.77-1.09 | **1.16**  **1.02-1.33** | **1.15**  **1.00-1.32** | 0.96  0.85-1.09 | 0.97  0.85-1.10 | 0.90  0.75-1.09 | 0.89  0.74-1.08 | **1.22**  **1.06-1.40** | **1.23**  **1.07-1.42** | 0.76  0.53-1.09 | 0.74  0.52-1.06 | 1.11  0.69-1.77 | 1.09  0.68-1.74 |
| **Years*Days** |  |  |  |  |  |  |  |  |  |  |  |  |  |  |  |  |  |  |
| 2017-19 by1^st^ | 1 | 1 | 1 | 1 | 1 | 1 | 1 | 1 | 1 | 1 | 1 | 1 | 1 | 1 | 1 | 1 | 1 | 1 |
| 2020 by2^nd^ | 0.95  0.67-1.34 | 0.94 0.66-1.33 | 1.09  0.82-1.47 | 1.08  0.80-1.46 | 0.75 0.40-1.43 | 0.75 0.39-1.43 | 0.71 0.43-1.17 | 0.72  0.43-1.19 | 1.28  0.82-2.01 | 1.29  0.81-2.03 | 1.49  0.74-2.98 | 1.52  0.76-3.05 | 1.45  0.97-2.16 | 1.37  0.92-2.06 | 0.83  0.15-4.53 | 0.83  0.15-4.54 | 2.18  0.67-7.08 | 2.40  0.73-7.91 |
| 2020 by3^rd^ | 0.96 0.77-1.20 | 0.95 0.76-1.19 | 1.13  0.94-1.37 | 1.11  0.92-1.34 | 0.94 0.62-1.43 | 0.93 0.61-1.42 | **0.68 0.49-0.93** | **0.70 0.51-0.97** | **1.34**  **1.00-1.79** | 1.34  0.99-1.80 | 1.44  0.92-2.26 | 1.43  0.91-2.24 | **1.66**  **1.23-2.25** | **1.64**  **1.21-2.22** | **2.57**  **1.09-6.07** | **2.57**  **1.09-6.09** | **4.08**  **1.74-9.55** | **4.61**  **1.92-11.05** |
| 2020 by4^th^ | **0.79 0.65-0.96** | **0.80 0.65-0.97** | 0.98  0.83-1.15 | 0.97  0.82-1.15 | 0.93 0.65-1.33 | 0.93 0.65-1.33 | **0.75 0.58-0.98** | **0.75 0.57-0.99** | 1.26  0.97-1.65 | 1.29  0.98-1.68 | **1.73**  **1.16-2.58** | **1.74**  **1.16-2.60** | **1.43**  **1.10-1.87** | **1.40**  **1.07-1.84** | 1.83  0.83-4.05 | 1.88  0.85-4.16 | **4.00**  **1.86-8.63** | **4.43**  **2.01-9.78** |
| 2021 by2^nd^ | 1.10 0.76-1.61 | 1.10 0.75-1.61 | 1.15  0.85-1.57 | 1.17  0.86-1.59 | 0.76 0.40-1.46 | 0.74 0.39-1.42 | 1.06 0.63-1.78 | 1.07 0.64-1.80 | 1.00  0.63-1.61 | 1.00  0.62-1.60 | 0.91  0.46-1.81 | 0.94  0.48-1.87 | 0.62  0.38-1.03 | **0.58**  **0.35-0.96** | 1.37  0.36-5.21 | 1.38  0.36-5.23 | 0.83  0.27-2.52 | 0.83  0.28-2.53 |
| 2021 by3^rd^ | 1.05 0.82-1.34 | 1.04 0.81-1.33 | 0.96  0.79-1.16 | 0.93  0.77-1.13 | 0.85 0.56-1.28 | 0.83 0.55-1.25 | 1.26 0.91-1.74 | 1.30 0.94-1.80 | 0.97 0.73-1.30 | 0.95  0.71-1.27 | 0.89  0.60-1.32 | 0.90  0.60-1.33 | 1.01  0.73-1.39 | 1.01  0.73-1.39 | 0.91  0.39-2.14 | 0.91  0.39-2.15 | 1.50  0.74-3.05 | 1.56  0.76-3.18 |
| 2021 by4^th^ | 0.92 0.75-1.13 | 0.90 0.74-1.11 | 1.02  0.87-1.20 | 1.01  0.86-1.19 | 1.01 0.73-1.42 | 1.01 0.72-1.41 | 0.98 0.74-1.29 | 0.99 0.75-1.31 | 1.05  0.82-1.35 | 1.04  0.81-1.33 | 0.93  0.67-1.29 | 0.94  0.67-1.31 | 1.08  0.83-1.41 | 1.08  0.82-1.41 | 0.92  0.46-1.86 | 0.96  0.47-1.93 | 1.21  0.66-2.21 | 1.23  0.67-2.25 |
| **Gender** |  |  |  |  |  |  |  |  |  |  |  |  |  |  |  |  |  |  |
| Male | 1 | 1 | 1 | 1 | 1 | 1 | 1 | 1 | 1 | 1 | 1 | 1 | 1 | 1 | 1 | 1 | 1 | 1 |
| Female | 1.00  0.95-1.05 | 1.00 0.95-1.06 | **1.25**  **1.20-1.31** | **1.24**  **1.19-1.30** | 0.95 0.87-1.04 | 0.95 0.87-1.04 | 1.03 0.96-1.11 | 1.03 0.96-1.11 | 0.97  0.91-1.04 | 0.97  0.91-1.04 | 1.06 0.96-1.17 | 1.06 0.96-1.16 | **0.78 0.73-0.84** | **0.79 0.74-0.85** | 0.96 0.79-1.17 | 0.95 0.78-1.16 | **1.22 1.06-1.41** | **1.22 1.06-1.41** |
| Unknown | 0.66  0.42-1.02 | - | 1.01  0.72-1.41 | **-** | 0.58  0.23-1.42 | - | 1.44  0.86-2.41 | - | 0.82  0.50-1.37 | - | 0.73  0.30-1.80 | - | 1.15  0.74-1.79 | **-** | - | - | 0.66  0.09-4.87 | **-** |
| **Age class (year)** |  |  |  |  |  |  |  |  |  |  |  |  |  |  |  |  |  |  |
| <1 | 1 | 1 | 1 | 1 | 1 | 1 | 1 | 1 | 1 | 1 | 1 | 1 | 1 | 1 | 1 | 1 | 1 | 1 |
| 1-5 | **1.43**  **1.20-1.71** | **1.44**  **1.21-1.72** | **1.63**  **1.37-1.93** | **1.63**  **1.37-1.94** | **1.60**  **1.11-2.30** | **1.59**  **1.10-2.28** | **1.46**  **1.23-1.72** | **1.45**  **1.22-1.71** | **1.33**  **1.10-1.60** | **1.32**  **1.10-1.59** | 0.87  0.68-1.23 | 0.87  0.68-1.12 | 1.07  0.87-1.33 | 1.07  0.87-1.33 | **2.67**  **1.31-5.40** | **2.65**  **1.31-5.38** | 0.78  0.59-1.03 | 0.78  0.59-1.03 |
| 6-19 | **1.45**  **1.19-1.77** | **1.45**  **1.19-1.78** | **3.05**  **2.53-3.67** | **3.05**  **2.53-3.68** | **1.69**  **1.13-2.52** | **1.68**  **1.12-2.50** | **0.54**  **0.43-0.67** | **0.52**  **0.42-0.65** | 1.21  0.98-1.51 | 1.22  0.98-1.51 | **2.00**  **1.51-2.64** | **2.01**  **1.52-2.65** | **2.35**  **1.86-2.96** | **2.35**  **1.87-2.97** | 1.64  0.76-3.56 | 1.67  0.77-3.62 | **0.50**  **0.35-0.73** | **0.50**  **0.34-0.73** |
| >19 | **2.07**  **1.74-2.47** | **2.08**  **1.74-2.48** | **4.36**  **3.67-5.19** | **4.39**  **3.69-5.22** | **2.72**  **1.89-3.91** | **2.70**  **1.88-3.89** | **0.30**  **0.25-0.36** | **0.29**  **0.24-0.35** | 1.17  0.97-1.40 | 1.17  0.97-1.41 | 1.00  0.77-1.29 | 1.00  0.77-1.29 | **1.99**  **1.61-2.46** | **1.99**  **1.61-2.46** | 0.64  0.31-1.35 | 0.65  0.31-1.37 | **0.19**  **0.13-0.26** | **0.18**  **0.13-0.25** |
| Unknown | **2.17**  **1.69-2.78** | - | **3.22**  **2.56-4.04** | - | 1.66  0.99-2.77 | - | **0.41**  **0.29-0.59** | - | **1.35**  **1.00-1.81** | - | 0.86  0.55-1.36 | - | **1.64**  **1.21-2.24** | - | 0.49  0.13-1.88 | - | **0.39**  **0.19-0.77** | - |
| **Circumstance** |  |  |  |  |  |  |  |  |  |  |  |  |  |  |  |  |  |  |
| I-Non occup | 1 | 1 | 1 | 1 | 1 | 1 | 1 | 1 | 1 | 1 | 1 | 1 | 1 | 1 | 1 | 1 | 1 | 1 |
| I-occup | 0.97  0.84-1.13 | 0.95 0.81-1.10 | **0.52 0.45-0.60** | **0.51**  **0.45-0.59** | 1.25 0.99-1.56 | 1.26  1.01-1.59 | **0.13 0.06-0.30** | **0.14 0.06-0.31** | **0.62**  **0.50-0.79** | **0.62**  **0.49-0.79** | **0.55**  **0.39-0.78** | **0.48**  **0.33-0.70** | **2.26 1.98-2.58** | **2.25 1.96-2.57** | 0.23 0.03-1.66 | 0.23 0.03-1.67 | 0.50 0.20-1.21 | 0.52 0.21-1.28 |
| Intentional | 1.05  0.97-1.14 | 1.06 0.97-1.15 | **1.53**  **1.44-1.62** | **1.52**  **1.43-1.61** | 1.34 1.18-1.52 | 1.33 1.17-1.51 | **1.49 1.29-1.72** | **1.53 1.32-1.77** | **0.52**  **0.46-0.60** | **0.52**  **0.45-0.59** | **0.55**  **0.46-0.66** | **0.56**  **0.47-0.67** | **0.72 0.65-0.80** | **0.71 0.64-0.79** | **4.70 3.44-6.44** | **4.59 3.35-6.30** | 1.06 0.78-1.44 | 1.08 0.79-1.47 |
| Covariates included per each product-specific model: Poison Center, Gender, Age class, Circumstance, Period_years, Period_days, The interaction term Years*Days | | | | | | | | | | | | | | | | | | |
