# Supplementary material for: The impact of COVID-19 management on the risk of exposure to specific chemical products: a multicentric Italian study based on 2017–2021 poison centers consultancies
Source: Front Public Health. 2025 Nov 27;13:1717130. doi: 10.3389/fpubh.2025.1717130 (PMC12695805; doi:10.3389/fpubh.2025.1717130)
Supplement: Supplementary file 3 [file Table_2.docx]

| **Table S2.** Crude and Adjusted ORs per each product-specific model (SIN-SEPI data from 2017 to 2021) | | | | | | | | | | | | | | | | | | |
| --- | --- | --- | --- | --- | --- | --- | --- | --- | --- | --- | --- | --- | --- | --- | --- | --- | --- | --- |
| **Variable** | **Cleaning products** | | | | | | **Detergents** | | | | **Biocides (BPR n.528/2012)** | | | | **Handwashing Cosmetics** | | | |
|  | **All-Purpose**  **(PC-CLN-2)**  OR  95%CI | | **Bleaching**  **(PC-CLN-3)**  OR  95%CI | | **Floor/stone/tile**  **(PC-CLN-12,13)**  OR  95%CI | | **Laundry**  **(PC-DET-1)**  OR  *95%CI* | | **Dishwashing**  **(PC-DET-3)**  OR  95%CI | | **For human**  **(PP-BIO-1)**  OR  95%CI | | **For surfaces**  **(PP-BIO-2)**  OR  95%CI | | **Soaps**  **(COSM-HS)**  OR  95%CI | | **Gel/Spray**  **(COSM-HGS)**  OR  95%CI | |
|  | Crude | Adjusted | Crude | Adjusted | Crude | Adjusted | Crude | Adjusted | Crude | Adjusted | Crude | Adjusted | Crude | Adjusted | Crude | Adjusted | Crude | Adjusted |
| *Sign. cut-off | p<.05 | p<.002 | p<.05 | p<.002 | p<.05 | p<.002 | p<.05 | p<.002 | p<.05 | p<.002 | p<.05 | p<.002 | p<.05 | p<.002 | p<.05 | p<.002 | p<.05 | p<.002 |
| Goodness-of-fit (H-L) | - | p=.637 | - | p<.001 | - | p=.158 | - | p=.040 | - | p=.068 | - | p=.755 | - | p=.139 | - | p=.410 | - | p=.057 |
| **Poison Center** |  |  |  |  |  |  |  |  |  |  |  |  |  |  |  |  |  |  |
| Pavia | 1 | 1 | 1 | 1 | 1 | 1 | 1 | 1 | 1 | 1 | 1 | 1 | 1 | 1 | 1 | 1 | 1 | 1 |
| Bergamo | 1.03  0.96-1.10 | 1.10  1.02-1.18  p=.011 | **0.65**  **0.61-0.70**  **p<.001** | **0.78**  **0.73-0.83**  **p<.001** | **0.74**  **0.65-0.85**  **p<.001** | 0.83  0.73-0.96  p=.009 | 1.08  0.98-1.18 | 0.90  0.82-0.99  p=.025 | **1.30**  **1.19-1.41**  **p<.001** | **1.21**  **1.11-1.32**  **p<.001** | **0.73**  **0.63-0.83**  **p<.001** | **0.71**  **0.61-0.81**  **p<.001** | 0.93  0.85-1.01 | 1.03  0.94-1.12 | 1.10  0.85-1.41 | 1.14  0.88-1.48 | **0.72**  **0.58-0.89**  **p=.003** | **0.62**  **0.50-0.76**  **p<.001** |
| Florence | 0.99  0.91-1.08 | 1.00  0.91-1.09 | **0.76**  **0.70-0.82**  **p<.001** | **0.79**  **0.73-0.85**  **p<.001** | **0.75**  **0.64-0.89**  **p<.001** | 0.79  0.67-0.93  p=.006 | 0.90  0.80-1.02 | 0.91  0.80-1.02 | 1.07 0.96-1.19 | 1.01  0.90-1.13 | 0.95  0.81-1.11 | 0.90  0.77-1.05 | 0.93  0.83-1.03 | 0.91  0.82-1.02 | 1.20  0.89-1.62 | 1.38  1.01-1.87  p=.040 | 0.88  0.69-1.13 | 0.84  0.66-1.08 |
| Foggia | **1.23**  **1.11-1.36**  **p<.001** | **1.21**  **1.09-1.34**  **p<.001** | 1.03  0.94-1.12 | 1.04  0.95-1.13 | 0.88  0.72-1.06 | 0.90  0.74-1.10 | 0.90  0.77-1.04 | 0.96  0.83-1.12 | **1.52**  **1.35-1.71**  **p<.001** | **1.45**  **1.28-1.63**  **p<.001** | **0.63**  **0.50-0.79**  **p<.001** | **0.59**  **0.47-0.75**  **p<.001** | **1.20**  **1.06-1.35**  **p=.005** | 1.13  0.99-1.28 | **0.45**  **0.25-0.81**  **p=.007** | 0.53  0.30-0.96  p=.035 | 1.18  0.90-1.54 | 1.09  0.83-1.43 |
| Naples | 0.92  0.82-1.02 | 0.90  0.80-1.01 | **1.26**  **1.16-1.36**  **p<.001** | **1.23**  **1.13-1.34**  **p<.001** | 1.17  0.99-1.39 | 1.23  1.03-1.46  p=.020 | 0.92  0.79-1.06 | 0.97  0.83-1.13 | 0.88  0.76-1.02 | 0.85  0.73-0.98  p=.028 | **0.64**  **0.51-0.80**  **p<.001** | **0.60**  **0.47-0.75**  **p<.001** | **0.71**  **0.61-0.83**  **p<.001** | **0.69**  **0.59-0.81**  **p<.001** | **0.45**  **0.25-0.80**  **p=.006** | 0.46  0.26-0.83  p=.010 | **0.43**  **0.28-0.65**  **p<.001** | **0.34**  **0.23-0.52**  **p<.001** |
| CHBG-Rome | **0.74**  **0.62-0.87**  **p<.001** | 0.93  0.78-1.10 | **0.57**  **0.50-0.66**  **p<.001** | 1.11  0.96-1.28 | **0.43**  **0.30-0.62**  **p<.001** | 0.64  0.44-0.93  p=.018 | **1.47**  **1.24-1.74**  **p<.001** | 0.85  0.72-1.01 | 1.14  0.95-1.36 | 0.99  0.82-1.19 | 1.03  0.80-1.33 | 0.98  0.76-1.27 | **0.72**  **0.59-0.89**  **p=.002** | 0.97  0.78-1.19 | 0.87  0.49-1.56 | 0.68  0.38-1.23 | **1.54**  **1.12-2.12**  **p=.008** | 0.81  0.59-1.12 |
| **Period years** |  |  |  |  |  |  |  |  |  |  |  |  |  |  |  |  |  |  |
| 2017-2019 | 1 | 1 | 1 | 1 | 1 | 1 | 1 | 1 | 1 | 1 | 1 | 1 | 1 | 1 | 1 | 1 | 1 | 1 |
| 2020 | **1.08**  **1.01-1.15**  **p=.025** | 1.25  1.05-1.50  p=.014 | 1.05  0.99-1.0 | 1.00  0.86-1.17 | **0.73**  **0.65-0.83**  **p<.001** | 0.77  0.56-1.07 | **0.82**  **0.75-0.90**  **p<.001** | 1.09  0.85-1.39 | 1.01  0.93-1.10 | 0.80  0.62-1.02 | **1.18**  **1.04-1.33**  **p=.008** | 0.77  0.53-1.12 | **1.48**  **1.37-1.60**  **p<.001** | 1.07  0.83-1.38 | 1.20  0.95-1.53 | 0.70  0.34-1.45 | **5.47**  **4.52-6.61**  **p<.001** | 1.46  0.69-3.12 |
| 2021 | **0.92**  **0.86-0.99**  **p=.017** | 0.97  0.80-1.17 | 1.03  0.98-1.09 | 1.00  0.86-1.16 | **0.88**  **0.78-0.99**  **p=.027** | 0.90  0.67-1.22 | **0.87**  **0.80-0.95**  **p=.002** | 0.85  0.66-1.09 | 0.99  0.91-1.07 | 0.98  0.79-1.23 | **1.27**  **1.13-1.43**  **p<.001** | 1.37  1.02-1.85  p=.040 | **1.10**  **1.01-1.19**  **p=.029** | 1.08  0.84-1.38 | 0.95  0.73-1.22 | 0.98  0.53-1.82 | **5.17**  **4.27-6.26**  **p<.001** | **4.34**  **2.51-7.60**  **p<.001** |
| **Period days** |  |  |  |  |  |  |  |  |  |  |  |  |  |  |  |  |  |  |
| 1^st^ (01/01 - 23/02) | 1 | 1 | 1 | 1 | 1 | 1 | 1 | 1 | 1 | 1 | 1 | 1 | 1 | 1 | 1 | 1 | 1 | 1 |
| 2^nd^ (24/02 - 10/03) | 1.08  0.93-1.25 | 1.07  0.88-1.31 | 1.01  0.90-1.13 | 0.97  0.83-1.15 | 1.08  0.85-1.37 | 1.21  0.90-1.62 | 1.04  0.85-1.26 | 1.10  0.86-1.42 | 1.06  0.88-1.27 | 1.02  0.80-1.29 | 0.90  0.68-1.19 | 0.84  0.57-1.22 | **1.72**  **1.45-2.04**  **p<.001** | **1.66**  **1.32-2.09**  **p<.001** | 0.78  0.44-1.36 | 0.74  0.36-1.53 | 1.43  0.92-2.24 | 1.25  0.55-2.82 |
| 3^rd^ (11/03 - 18/05) | 1.04  0.95-1.15 | 1.04  0.92-1.18 | 1.03  0.96-1.11 | 1.04  0.94-1.15 | 0.87  0.74-1.01 | 0.92  0.75-1.12 | 0.95  0.84-1.08 | 0.96  0.82-1.13 | **1.13**  **1.01-1.26**  **p=.042** | 1.06  0.91-1.23 | 1.07  0.91-1.26 | 1.04  0.83-1.30 | **1.20**  **1.06-1.36**  **p=.003** | 1.04  0.88-1.23 | 1.00  0.72-1.38 | 0.83  0.54-1.27 | **1.77**  **1.32-2.37**  **p<.001** | 0.94  0.53-1.66 |
| 4^th^ (19/05 – 31/12) | 1.04  0.96-1.13 | 1.12  1.01-1.25  p=.034 | 0.96  0.90-1.02 | 0.99  0.91-1.08 | 0.89  0.78-1.02 | 0.92  0.77-1.08 | 1.09  0.99-1.21 | 1.15  1.00-1.32  p=.046 | 1.04  0.94-1.14 | 0.97  0.85-1.10 | 0.98  0.85-1.13 | 0.89  0.74-1.08 | **1.37**  **1.24-1.53**  **p<.001** | 1.23  1.07-1.42  p=.004 | 0.82  0.62-1.08 | 0.74  0.52-1.06 | **1.82**  **1.40-2.36**  **p<.001** | 1.09  0.68-1.74 |
| **Years*Days** |  |  |  |  |  |  |  |  |  |  |  |  |  |  |  |  |  |  |
| 2017-19 by1^st^ | 1 | 1 | 1 | 1 | 1 | 1 | 1 | 1 | 1 | 1 | 1 | 1 | 1 | 1 | 1 | 1 | 1 | 1 |
| 2020 by2^nd^ | 1.16  0.90-1.49 | 0.94  0.66-1.34 | 1.10  0.89-1.35 | 1.08  0.80-1.46 | 0.78  0.47-1.29 | 0.75  0.39-1.43 | 0.76  0.52-1.12 | 0.72  0.43-1.19 | 1.04 0.75-1.43 | 1.29  0.81-2.03 | 1.06  0.65-1.72 | 1.52  0.76-3.05 | **2.18**  **1.69-2.80**  **p<.001** | 1.37  0.92-2.06 | 0.52  0.13-2.07 | 0.83  0.15-4.54 | **3.50**  **1.89-6.45**  **p<.001** | 2.40  0.73-7.91 |
| 2020 by3^rd^ | **1.15**  **1.02-1.30**  **p=.021** | 0.95  0.76-1.19 | **1.18 1.07-1.30**  **p<.001** | 1.11  0.92-1.34 | **0.72**  **0.57-0.92**  **p=.008** | 0.93  0.61-1.42 | **0.68**  **0.56-0.82**  **p<.001** | 0.70  0.51-0.97  p=.032 | **1.17 1.01-1.35**  **p=.034** | 1.34  0.99-1.80 | 1.18  0.95-1.47 | 1.43  0.91-2.24 | **1.51**  **1.32-1.74**  **p<.001** | **1.64**  **1.21-2.22**  **p=.002** | **1.68**  **1.15-2.44**  **p=.007** | 2.57  1.09-6.09  p=.031 | **4.88**  **3.72-6.39**  **p<.001** | **4.61**  **1.92-11.05**  **p<.001** |
| 2020 by4^th^ | 1.02  0.95-1.11 | 0.80  0.65-0.97  p=.024 | 0.98 0.92-1.05 | 0.97  0.82-1.15 | **0.71**  **0.61-0.83**  **p<.001** | 0.93  0.65-1.33 | **0.87**  **0.78-0.97**  **p=.011** | 0.75  0.57-0.99  p=.039 | 1.01 0.91-1.12 | 1.29  0.98-1.68 | **1.24**  **1.08-1.43**  **p=.002** | 1.74  1.16-2.60  p=.007 | **1.56**  **1.43-1.71**  **p<.001** | 1.40  1.07-1.84  p=.015 | 1.15  0.86-1.54 | 1.88  0.85-4.16 | **5.66**  **4.67-6.85**  **p<.001** | **4.43**  **2.01-9.78**  **p<.001** |
| 2021 by2^nd^ | 1.06  0.79-1.41 | 1.10  0.75-1.61 | 1.17 0.93-1.46 | 1.17  0.86-1.59 | 0.89  0.53-1.49 | 0.74  0.39-1.42 | 0.90  0.61-1.32 | 1.07  0.64-1.80 | 1.00 0.70-1.43 | 1.00  0.62-1.60 | 1.13  0.67-1.90 | 0.94  0.48-1.87 | 0.89  0.60-1.32 | 0.58  0.35-0.96  p=.034 | 1.25  0.46-3.38 | 1.38  0.36-5.23 | **3.46**  **1.76-6.79**  **p<.001** | 0.83  0.28-2.53 |
| 2021 by3^rd^ | 0.96  0.84-1.10 | 1.04  0.81-1.33 | 0.98 0.88-1.10 | 0.93  0.77-1.13 | **0.74**  **0.57-0.96**  **p=.023** | 0.83  0.55-1.25 | 0.98  0.82-1.16 | 1.30  0.94-1.80 | 1.01 0.86-1.20 | 0.95  0.71-1.27 | **1.31**  **1.05-1.65**  **p=.019** | 0.90  0.60-1.33 | 0.94  0.78-1.13 | 1.01  0.73-1.39 | 0.90  0.53-1.55 | 0.91  0.39-2.15 | **5.04**  **3.79-6.70**  **p<.001** | 1.56  0.76-3.18 |
| 2021 by4^th^ | **0.90**  **0.83-0.98**  **p=.015** | 0.90  0.74-1.11 | 1.03 0.97-1.10 | 1.01  0.86-1.19 | 0.90  0.79-1.04 | 1.01  0.72-1.41 | **0.88**  **0.79-0.97**  **p=.013** | 0.99  0.75-1.31 | 1.00 0.90-1.10 | 1.04  0.81-1.33 | **1.21**  **1.06-1.39**  **p=.006** | 0.94  0.67-1.31 | **1.22**  **1.11-1.34**  **p<.001** | 1.08  0.82-1.41 | 0.87  0.64-1.20 | 0.96  0.47-1.93 | **4.66**  **3.83-5.67**  **p<.001** | 1.23  0.67-2.25 |
| **Gender** |  |  |  |  |  |  |  |  |  |  |  |  |  |  |  |  |  |  |
| Male | 1 | 1 | 1 | 1 | 1 | 1 | 1 | 1 | 1 | 1 | 1 | 1 | 1 | 1 | 1 | 1 | 1 | 1 |
| Female | 1.05  0.99-1.11 | 1.00  0.95-1.06 | **1.39**  **1.33-1.45**  **p<.001** | **1.24**  **1.19-1.30**  **p<.001** | 1.00  0.92-1.10 | 0.95  0.87-1.04 | **0.88**  **0.82-0.94**  **p<.001** | 1.03  0.96-1.11 | 0.95  0.89-1.02 | 0.97  0.91-1.04 | 1.07  0.98-1.18 | 1.06  0.96-1.16 | **0.83**  **0.78-0.89**  **p<.001** | **0.79**  **0.74-0.85**  **p<.001** | 0.92  0.76-1.12 | 0.95  0.78-1.16 | 1.06  0.92-1.22 | 1.22  1.06-1.41  p=.006 |
| **Age class (year)** |  |  |  |  |  |  |  |  |  |  |  |  |  |  |  |  |  |  |
| <1 | 1 | 1 | 1 | 1 | 1 | 1 | 1 | 1 | 1 | 1 | 1 | 1 | 1 | 1 | 1 | 1 | 1 | 1 |
| 1-5 | **1.44**  **1.21-1.72**  **p<.001** | **1.44**  **1.21-1.72**  **p<.001** | **1.66**  **1.39-1.98**  **p<.001** | **1.63**  **1.37-1.94**  **p<.001** | **1.63**  **1.13-2.35**  **p=.009** | 1.59  1.10-2.28  p=.013 | **1.45**  **1.23-1.72**  **p<.001** | **1.45**  **1.22-1.71**  **p<.001** | **1.31**  **1.09-1.58**  **p=.004** | 1.32  1.10-1.59  p=.003 | 0.87  0.67-1.12 | 0.87  0.68-1.12 | 1.08  0.88-1.34 | 1.07  0.87-1.33 | **2.61**  **1.29-5.29**  **p=.008** | 2.65  1.31-5.38  p=.007 | 0.77  0.59-1.02 | 0.78  0.59-1.03 |
| 6-19 | **1.46**  **1.20-1.79**  **p<.001** | **1.45**  **1.19-1.78**  **p<.001** | **3.66**  **3.04-4.40**  **p<.001** | **3.05**  **2.53-3.68**  **p<.001** | **1.93**  **1.29-2.86**  **p=.001** | 1.68  1.12-2.50  p=.012 | **0.61**  **0.49-0.75**  **p<.001** | **0.52**  **0.42-0.65**  **p<.001** | 1.02  0.82-1.26 | 1.22  0.98-1.51 | **1.78**  **1.36-2.35**  **p<.001** | **2.01**  **1.52-2.65**  **p<.001** | **2.13**  **1.70-2.68**  **p<.001** | **2.35**  **1.87-2.97**  **p<.001** | **3.17**  **1.50-6.68**  **p=.002** | 1.67  0.77-3.62 | **0.55**  **0.39-0.78**  **p<.001** | **0.50**  **0.34-0.73**  **p<.001** |
| >19 | **2.10**  **1.76-2.50**  **p<.001** | **2.08**  **1.74-2.48**  **p<.001** | **4.92**  **4.14-5.84**  **p<.001** | **4.39**  **3.69-5.22**  **p<.001** | **3.09**  **2.16-4.43**  **p<.001** | **2.70**  **1.88-3.89**  **p<.001** | **0.31**  **0.26-0.38**  **p<.001** | **0.29**  **0.24-0.35**  **p<.001** | 1.01  0.84-1.21 | 1.17  0.97-1.41 | 0.89  0.69-1.14 | 1.00  0.77-1.29 | **1.96**  **1.59-2.42**  **p<.001** | **1.99**  **1.61-2.46**  **p<.001** | 1.07  0.52-2.20 | 0.65  0.31-1.37 | **0.20**  **0.15-0.27**  **p<.001** | **0.18**  **0.13-0.25**  **p<.001** |
| **Circumstance** |  |  |  |  |  |  |  |  |  |  |  |  |  |  |  |  |  |  |
| I-Non occup | 1 | 1 | 1 | 1 | 1 | 1 | 1 | 1 | 1 | 1 | 1 | 1 | 1 | 1 | 1 | 1 | 1 | 1 |
| I-occup | **1.17**  **1.01-1.35**  **p=.043** | 0.95  0.81-1.10 | **0.80**  **0.70-0.92**  **p=.002** | **0.51**  **0.45-0.59**  **p<.001** | **1.73**  **1.38-2.16**  **p<.001** | 1.26  1.01-1.59  p=.044 | **0.05**  **0.02-0.11**  **p<.001** | **0.14**  **0.06-0.31**  **p<.001** | **0.59**  **0.47-0.74**  **p<.001** | **0.62**  **0.49-0.79**  **p<.001** | **0.50**  **0.34-0.73**  **p<.001** | **0.48**  **0.33-0.70**  **p<.001** | **3.07**  **2.69-3.49**  **p<.001** | **2.25**  **1.96-2.57**  **p<.001** | **0.09**  **0.01-0.66**  **p=.017** | 0.23  0.03-1.67 | **0.20**  **0.08-0.47**  **p<.001** | 0.52  0.21-1.28 |
| Intentional | **1.21**  **1.12-1.31**  **p<.001** | 1.06  0.97-1.15 | **2.38**  **2.25-2.52**  **p<.001** | **1.52**  **1.43-1.61**  **p<.001** | **1.74**  **1.54-1.96**  **p<.001** | **1.33**  **1.17-1.51**  **p<.001** | **0.60**  **0.53-0.68**  **p<.001** | **1.53**  **1.32-1.77**  **p<.001** | **0.47**  **0.42-0.54**  **p<.001** | **0.52**  **0.45-0.59**  **p<.001** | **0.71**  **0.60-0.83**  **p<.001** | **0.56**  **0.47-0.67**  **p<.001** | 0.94  0.85-1.05 | **0.71**  **0.64-0.79**  **p<.001** | **2.22**  **1.76-2.79**  **p<.001** | **4.59**  **3.35-6.30**  **p<.001** | **0.56**  **0.43-0.73**  **p<.001** | 1.08  0.79-1.47 |
| ***In bold the significant p-values: Crude ORs (p<.05) and Adjusted ORs (threshold at p=.002 after Bonferroni correction).**  H-L: Hosmer & Lemeshow test  Covariates included per each product-specific model: Poison Center, Gender, Age class, Circumstance, Period_years, Period_days, The interaction term Years*Days | | | | | | | | | | | | | | | | | | |
